# Bone trait ranking in the population is not established during antenatal growth but is robustly established in the first postnatal year

**DOI:** 10.1371/journal.pone.0203945

**Published:** 2018-09-17

**Authors:** Lise Skåren, Xiaofang Wang, Åshild Bjørnerem

**Affiliations:** 1 Department of Clinical Medicine, UiT The Arctic University of Norway, Tromsø, Norway; 2 Endocrine Centre, Austin Health, University of Melbourne, Melbourne, Australia; 3 Department of Obstetrics and Gynaecology, University Hospital of North Norway, Tromsø, Norway; TNO, NETHERLANDS

## Abstract

Efforts to understand the pathophysiology of bone fragility must focus on bone traits during growth. We hypothesized that variance in individual trait ranking in the population distribution is established by genetic factors and is reflected in foetal trait ranking in early pregnancy, but intrauterine factors modify trait ranking in late pregnancy, followed by the reinstating of this ranking during the first postnatal year. Thus, relations with paternal factors are present in early pregnancy but are then lost and subsequently reinstated postnatal. We recruited 399 healthy pregnant women aged 20–42 years from The Mercy Hospital for Woman in Melbourne, Australia. Foetal femur length (FL) and knee-heel length (KHL) were measured by ultrasound during gestation, and FL, KHL, body length and weight were measured in neonates, infants, and parents. The z-scores were calculated using Royston models. Pearson correlation was used to assess tracking and linear mixed models to test the associations. Correlations between FL and KHL z-scores of the same trait at 20 and 30 weeks gestation, at birth, and at 12 and 24 months of age (r = 0.1–0.3) and of body length and weight at birth, and 6, 12 and 24 months (r = 0.3–0.5) became more robust after 6–12 months (r = 0.4–0.8). FL and KHL z-scores at 20 weeks gestation accounted for 4–5% of total variance, while FL, KHL, body length and weight z-scores at birth accounted for 13–26% of total variance in the same traits at 24 months. Maternal FL and KHL were associated with foetal FL and KHL at 20 and 30 weeks, but there were no such associations for paternal FL and KHL with foetal traits during gestation. Both maternal and paternal traits were associated with infant traits. Tracking in traits is not established antenatal but is robustly established at 6–12 months of age.

## Introduction

Low birth weight and poor growth in infancy are associated with increased rates of chronic disease and osteoporosis in adulthood [1–3]. Efforts to understand the pathophysiology of bone fragility must focus not only on age-related bone loss but also on bone size attained during growth [4]. Genetic factors account for most of the variance in traits such as height and bone dimensions in adulthood [5,6]. However, it is uncertain whether a trait is assigned its individual ranking in the population distribution during intrauterine life or early infancy [7]. Most, but not all, studies suggest that adult dimensions are predicted by dimensions at birth [8,9]. If this is correct, then genetic and environmental factors operating during intrauterine life should predict dimensions both at birth and in adulthood. Several studies suggest otherwise. Bjørnerem et al. reported little tracking during intrauterine life and that the ranking of femur length in early gestation predicted only 10% of the variance in trait ranking at the end of gestation [10]. In addition, Wang et al. reported that height at 6 months and weight at 12 months of age, but not at birth, predicted bone dimensions in females at 18 years of age [11]. The latter observations challenge the notion that tracking is well established during intrauterine growth and favour the establishment of individual trait ranking in the population within the first 6–12 months of postnatal life.

There are few longitudinal studies of antenatal and postnatal growth, and birth weight is often used as a proxy for antenatal growth [7]. However, it does not provide information on growth patterns during the stages of *in utero* growth. There is considerable evidence that the velocity of foetal length and weight growth slows down from approximately 30–34 weeks gestation [12]. Maternal constraints are the major non-genetic factor that determine foetal growth at term 13]; such constraints include maternal and uteroplacental physiological factors that limit foetal growth by limiting nutrient supply to the foetus and thus override the genetically determined growth of the foetus [13]. This influence on foetal growth may be present in all pregnancies but particularly influential in younger mothers, nulliparous women, those with small maternal size, and those carrying multiple pregnancies [13]. This mechanism of reducing the growth velocity allows a genetically large child to be delivered successfully, and foetal growth that is held up during the end of pregnancy is often followed by catch-up growth after delivery [12]. Rapid growth following a period of growth restriction illustrates the tendency to return to the original growth path or trajectory if it has been altered by illness or other circumstances [12]. The weak correlation (0.3) between birth length and adult height but the strong correlation (0.8) between length at 2 years of age and adult height reflect the maternal control of new-born size [12,13]. That foal birth size followed maternal size in experiments crossing a Shetland pony with a Shire horse suggests that growth of the offspring is limited by the mother before birth and returns to its genetically determined trajectory after birth [14]. If maternal constraints operate over the whole range of foetal sizes in humans and push growth off the genetically determined path during late gestation, a correlation between a foetal trait z-score during early gestation (20 weeks) should be lost during later gestation (30 weeks) and reinstated after birth. If so, robust correlations between trait ranking at 20 weeks gestation and the same trait ranking at 6 to 12 months should be observed.

However, it is plausible that the ranking of a trait in adulthood is largely determined by genetic factors, and this notion would be supported if trait ranking in the second trimester (20 weeks) does not predict trait ranking at birth (because of maternal constraint) but does predict trait ranking at 6, 12 and 24 months. If this is the case, then an individual’s trait ranking in the population distribution could be largely determined by genetic factors. If this is not the case, then intrauterine and postnatal environmental factors could be more important in determining the variance in an individual’s trait ranking.

While the maternal influence on foetal growth is well known, the role of paternal factors in foetal growth is not known [13]. Moreover, it is well documented that both parents’ proportions predict postnatal growth, but the effects of maternal and paternal proportions on trait tracking and variation in offspring antenatal growth traits are less clear [15,16].

The aims of this study were to determined femur length (FL) and knee-heel length (KHL) prenatal (20 and 30 weeks gestation), at birth and postnatal (6, 12 and 24 months old) to investigate whether trait ranking in the second trimester and at birth predicts trait ranking of offspring and the contribution of parents to the variance in antenatal and postnatal trait ranking of offspring. We hypothesized that the variance in individual trait ranking in the population distribution is established by genetic factors, as reflected in foetal trait ranking in early pregnancy, but that intrauterine factors modify trait ranking in late pregnancy due to maternal constraints, although it is reinstated during the first year of postnatal life. Thus, the relations with paternal dimensions are present in early pregnancy, lost and then reinstated only after six months of postnatal life.

## Materials and methods

### Subjects

**Fig 1:**
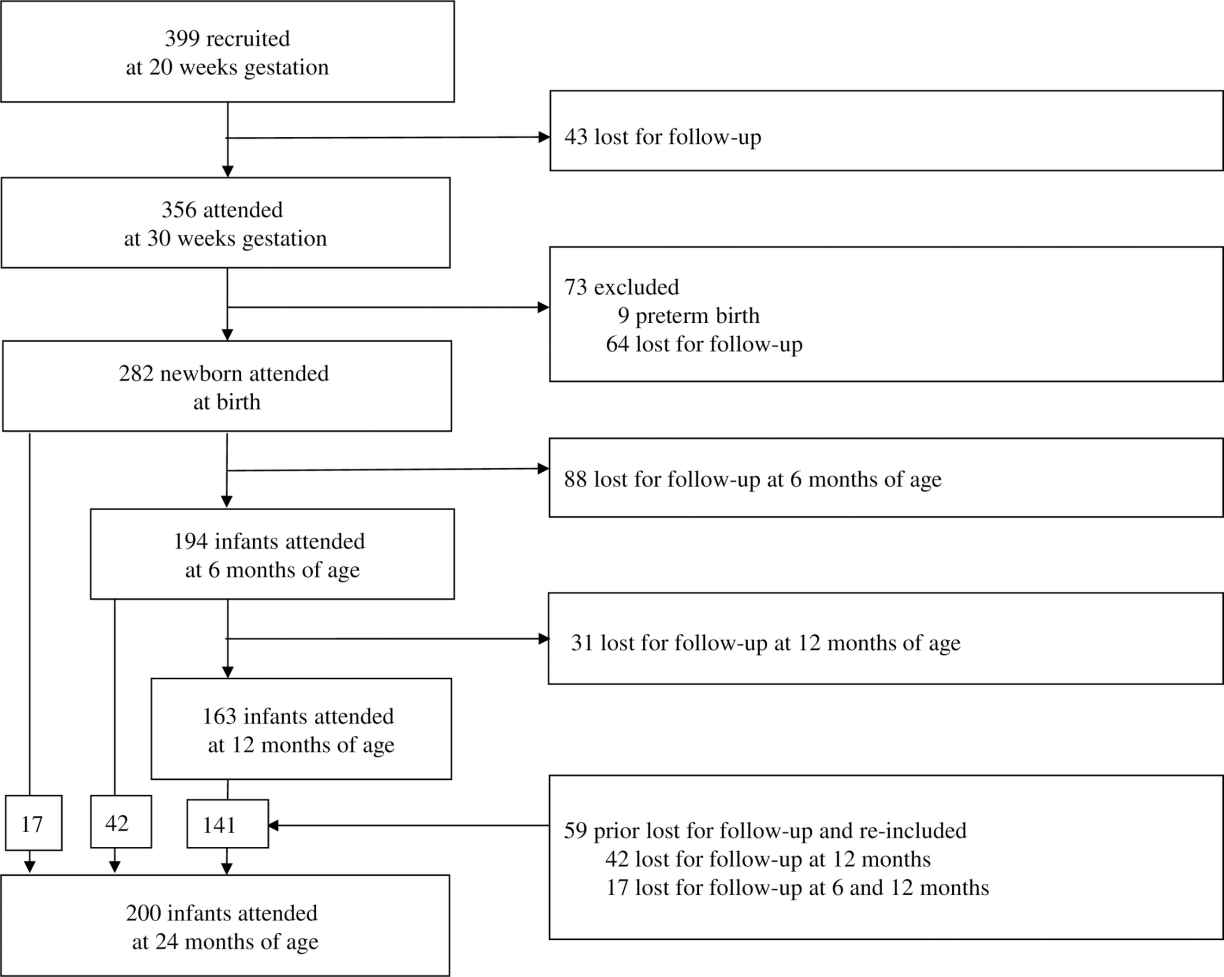
Participating pregnant women and offspring who remained at each time point.

Between July 2008 and June 2009, 399 healthy pregnant women aged 20–42 years with a single normal foetus were recruited at their 20-week-gestation routine ultrasound scan at The Mercy Hospital for Women in Melbourne, Australia (Fig 1). Among them, 43 were lost to follow-up, 356 were willing to have an additional ultrasound scan at 30 weeks gestation, and 345 of the husbands or partners were willing to participate. After birth, 282 term new-borns were available for measurements, 194 were willing to come for measurements at 6 months, 163 were willing to come at 12 months, and 200 were willing to come at 24 months. All participants gave written informed consent. Mercy Health & Aged Care Human Research Ethics Committee approved the study.

## Methods

Gestation was determined based on the last menstrual period unless the gestational age based on the first ultrasound measurement (crown-rump length (CRL) before 12 weeks or biparietal diameter (BPD) at 12–20 weeks) differed by more than 7 days; in such cases, gestational age was based on ultrasound assessments. Foetal growth was monitored using 2D ultrasound assessments of FL and KHL at 2 occasions; at 20 (range 17–23) and 30 (range 27–34) weeks gestation. Measurements were obtained by two experienced ultrasonographers using a Philips IU22, Philips HDI-5000 or a Philips HDI-300 ultrasound machine. We excluded foetuses who had major malformations detected by ultrasound scan or who were delivered preterm before 37 weeks gestation. A questionnaire included maternal lifestyle such as current smoking and alcohol use, parity, and country of birth to classify their ethnicity. Of the 370 women who completed the questionnaires, 279 (75.4%) reported that they were Caucasians, while 24.6% were of different multi-ethnic origins, mainly from Asian countries.

Following birth (1 to 7 days of age) and at 6, 12 and 24 months of age, the weight, crown-heel length (CHL), KHL and FL were measured by two trained researchers. CHL was measured to the nearest 0.1 cm using a lengthboard (Ellard Instrumentation Ltd., Seattle, WA), and KHL and FL were measured using a hand-held BK5 infant knemometer (Force Technology, Brondby, Denmark) [17]. Birth weight was measured on regularly calibrated scales. Parental standing height was measured to the nearest 0.1 cm using a Holtain stadiometer fixed on the wall, and weight was measured to the nearest 0.1 kg by an electronic scale while they were wearing light clothing without shoes at 30 weeks gestation. Parental body mass index (BMI) was calculated as weight divided by height^2^ (kg/m^2^). FL was determined to the nearest 0.5 cm as the distance from the greater trochanter to the lateral condyle [18], and KHL was measured to the nearest 0.1 cm using a hand-held knemometer.

All variables were checked for normality by visual inspection of the histograms. We performed non-response analysis and compared the characteristics of those with and without measurements at 24 months of age. Royston models were fitted to foetal and infant growth measurements to create z-scores for size measurements during growth [19]. Pearson correlation was used to assess tracking between FL and KHL z-scores at 20 and 30 weeks of gestation, at birth and at 6, 12 and 24 months and between body length and weight in neonates and infants at 6, 12 and 24 months. Linear mixed models were used to explore the effect of foetal FL and KHL at 20 weeks of gestation; neonatal FL, KHL, body length and weight; and parental traits on the same traits in infants at 12 and 24 months of age. Maternal age, smoking (no vs. yes), alcohol intake during gestation (no vs. yes), primiparous (no vs. yes), Caucasian ethnicity (no vs. yes), parental weight and offspring sex were considered as covariates. The p-value for entering variables was < 0.25, and that for deleting variables was > 0.15 [20]. Linear regression models were used to assess the variance in the outcome explained by each of the exposure variables by calculating the change in R^2^ before and after inclusion of each of the factors in the models. The SAS software version 9.4 (SAS Institute, Inc, Cary, NC, USA) was used for data analyses.

## Results

**Fig 2:**
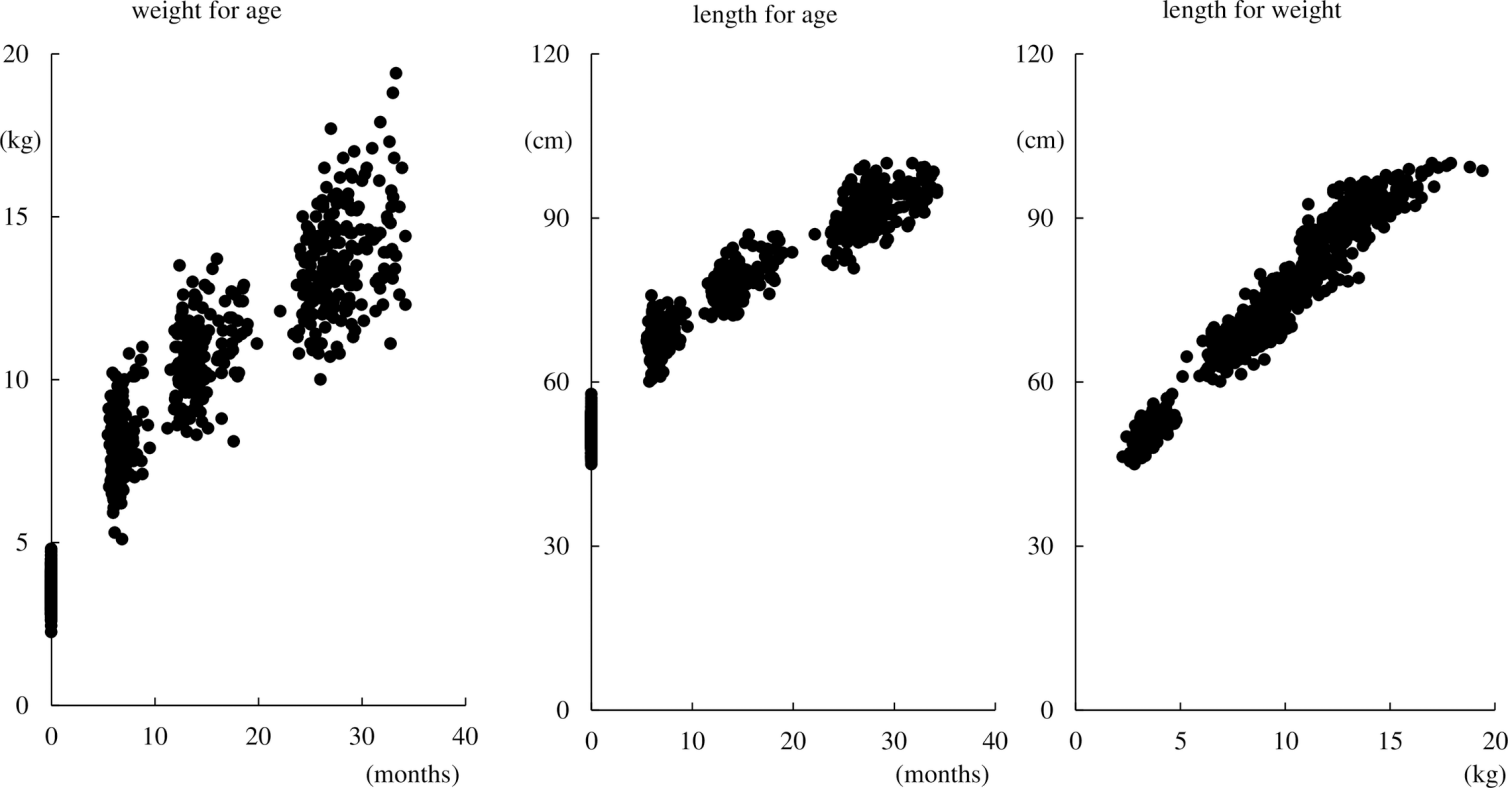
Growth charts showing weight-for-age, length-for-age and length-for-weight for the offspring.

Characteristics of the participants are shown in Table 1. The mean maternal age was 31.3 years, and 75.4% were of Caucasian ethnicity, 46.8% were primiparous and the mean paternal age was 33.8 years. The offspring were measured twice in gestation, at a mean age of 19.9 and 30.5 weeks, as newborn and three times after birth at a mean age of 6.6, 14.4 and 28.0 months, respectively. The weight-for-age, length-for-age and length-for-weight measurements showed that the offspring in this study were growing healthy (Fig 2). In the non-response analysis, mothers of offspring with measurements at 24 months of age more often were Caucasians (81.5% vs. 57.6%), were taller (165.1 vs. 163.3 cm) and had taller partners (178.6 vs. 176.6 cm) (all p < 0.05), while their age, weight, parity and smoking were not different.

**Table 1:** Characteristics of the mothers, fathers and offspring during gestation and after birth.

	n	Mean (SD)		Range	n	Mean (SD)	Range
Parents			Mothers			Fathers	
Age (years)	370		31.3 (4.5)	20–42	318	33.8 (5.7)	22–59
Height (cm)	370		164.3 (6.7)	145–188	345	177.7 (7.2)	158–199
Weight (kg)	370		76.9 (15.5)	46–140	345	86.9 (14.1)	48–131
Body mass index (kg/m^2^)	370		28.5 (5.2)	19–49	345	27.5 (4.1)	17–43
Femur length (cm)	370		41.3 (2.9)	34–49	344	43.5 (3.0)	36–55
Knee-heel length (cm)	370		51.1 (2.8)	41–61	344	55.5 (2.7)	47–64
Caucasian ethnicity, n (%)	370		279 (75.4)				
Primiparous, n (%)	355		166 (46.8)				
Smoking, n (%)	356		30 (8.4)				
Alcohol, n (%)	354		74 (20.9)				
**Fetuses**			**20 weeks**			**30 weeks**	
Gestational age (weeks)	399		19.9	17–23	356	30.5	27–34
Femur length (cm)	399		3.2 (0.2)	2.3–4.1	356	5.8 (0.3)	5.0–6.8
Knee heel length (cm)	387		5.7 (0.5)	4.2–7.2	353	9.9 (0.7)	8.2–12.1
**Infants**			**Neonates**			**6 months**	
Age	282		39.7 (1.2)^a^	37–42	194	6.6 (0.7)^b^	5.5–9.5
Body length (cm)	282		51.1 (2.1)	46–58	194	68.0 (2.9)	60–75
Body weight (kg)	282		3.5 (0.5)	2.3–4.8	194	8.1 (1.0)	5–11
Femur length (cm)	280		13.0 (0.9)	10–17	193	18.3 (1.0)	15–22
Knee heel length (cm)	282		12.8 (0.6)	11–15	194	17.7 (0.9)	15–21
			**12 months**			**24 months**	
Age (months)	163		14.4 (1.9)	11–19	200	28.0 (2.8)	20–34
Body length (cm)	163		78.5 (3.2)	72–87	200	91.4 (4.1)	81–100
Body weight (kg)	163		10.6 (1.2)	8–14	199	13.7 (1.7)	10–19
Femur length (cm)	163		22.1 (1.1)	19–25	200	26.4 (1.8)	23–32
Knee heel length (cm)	163		21.2 (1.2)	19–25	200	26.1 (1.5)	22–31

Parental proportions were measured at 30 weeks gestation. Information concerning maternal current smoking (no vs. yes), alcohol intake in the pregnancy (no vs. yes), primiparous (no vs. yes) and Caucasian ethnicity (no vs. yes) was obtained through questionnaires.

^a^weeks

^b^months

Correlations between FL z-scores at 20 weeks gestation with those at birth, 6, 12 and 24 months were between 0.1 and 0.3 (Table 2). Similar patterns were seen with KHL z-scores at 20 weeks gestation and later. The most robust correlations occurred after birth (ranging from 0.4 to 0.7, p < 0.001), particular after 6 months, and correlations in KHL z-scores between 6 and 12 months and between 12 and 24 months were approximately 0.7 (all p < 0.001). The observations were similar for body length and body weight z-scores, with moderate correlations between birth and 6, 12 and 24 months (ranging from 0.4 to 0.5) and strong correlations between z-scores at 6 months and later (ranging from 0.7 to 0.8, all p < 0.001). Multi- vs. primiparous pregnancies correlated with foetal KHL at 30 weeks gestation (r = 0.13, p = 0.018) and at birth (r = 0.15, p = 0.011) but not with foetal FL. Maternal age, paternal age and ethnicity were not correlated with foetal KHL or FL.

**Table 2:** Correlation between same traits z-scores in offspring across gestation, birth and infancy and parental same traits.

	At 30 weeks gestation	At birth	At 6 months	At 12 months	At 24 months	Maternal	Paternal
**Femur Length**							
At 20 weeks gestation	0.49***	0.31***	0.18*	0.06	0.21**	0.15**	0.03
At 30 weeks gestation		0.27***	0.24**	0.12	0.36***	0.13*	0.09
At birth			0.30***	0.24**	0.34***	0.15*	0.03
At 6 months				0.43***	0.41***	0.04	0.05
At 12 months					0.46***	0.13	0.11
At 24 months						0.15*	0.12
**Knee Heel Length**							
Gestation wk 20	0.25***	0.35***	0.21**	0.28***	0.22**	0.11*	0.09
Gestation wk 30		0.34***	0.21**	0.11	0.10	0.13*	0.12*
At birth			0.51***	0.43***	0.45***	0.24***	0.28***
At 6 months				0.68***	0.61***	0.26***	0.25***
At 12 months					0.72***	0.21**	0.22**
At 24 months						0.26***	0.32***
**Body length**							
At birth			0.49***	0.52***	0.51***	0.27***	0.23***
At 6 months				0.82***	0.74***	0.22***	0.28***
At 12 months					0.81***	0.32***	0.30***
At 24 months						0.37***	0.33***
**Body weight**							
At birth			0.36***	0.37***	0.38***	0.31***	0.10
At 6 months				0.74***	0.66***	0.27***	0.15*
At 12 months					0.84***	0.16*	0.08
At 24 months						0.30***	0.24***

Values are Pearson correlation coefficients.

*p < 0.05

** p < 0.01

*** p < 0.001

Maternal FL was associated with foetal FL at 20 and 30 weeks gestation (β = 0.14 [95% confidence interval 0.04, 0.24] and β = 0.15 [0.05, 0.25]), but paternal FL was not (β = 0.02 [-0.08, 0.12] and β = 0.08 [-0.03, 0.19]) (Table 3). Similarly, maternal KHL was associated with foetal KHL at 20 and 30 weeks gestation (β = 0.11 [0.01, 0.21] and β = 0.20 [0.06, 0.34]), but there were no such associations for paternal KHL (β = 0.10 [0.00, 0.20] and β = 0.07 [-0.05, 0.19]). The contribution of maternal FL to variance in foetal FL was 3% at 20 weeks and 2% at 30 weeks gestation. The contribution of maternal KHL to variance in foetal KHL was 1% at 20 weeks and 1% at 30 weeks gestation. There was no independent contribution of paternal traits to the corresponding foetal traits during gestation.

**Table 3:** Effect of parental femur length and knee heel length (exposure) on the same trait of offspring at 20 weeks and 30 weeks gestation (outcomes).

	At 20 weeks gestation		At 30 weeks gestation	
	**Fetal femur length (FL)**			
	Estimates (95% CI)^a^	Estimates (95% CI)^b^	Estimates (95% CI)^a^	Estimates (95% CI)^b^
**Maternal FL (SD)**	**0.14 (0.04, 0.24)**	**0.14 (0.02, 0.26)**	**0.15 (0.05, 0.25)**	0.06 (-0.06, 0.18)
**Paternal FL (SD)**	0.02 (-0.08, 0.12)	-0.02 (-0.14, 0.10)	0.08 (-0.03, 0.19)	0.07 (-0.05, 0.19)
	**Fetal knee heel length (KHL)**			
**Maternal KHL (SD)**	**0.11 (0.01, 0.21)**	0.09 (-0.03, 0.21)	**0.14 (0.04, 0.24)**	**0.20 (0.06, 0.34)**
**Paternal KHL (SD)**	0.10 (0.00, 0.20)	0.08 (-0.04, 0.20)	**0.12 (0.01, 0.23)**	0.07 (-0.05, 0.19)

Values are β-estimates (95% confidence interval (CI)) per standard deviation (SD) unit change in parental traits.

Estimates are presented ^a^unadjusted and ^b^adjusted mutually for maternal and paternal same trait, maternal age, current smoking (no vs. yes), alcohol intake (no vs. yes), primiparous (no vs. yes) and Caucasians vs. other ethnicity, maternal and paternal weight, fetal sex (male vs. female) in linear regression analysis, and the significant results are shown in bold. We used p-value < 0.25 for entering variables and p-value > 0.15 for deleting variable.

**Fig 3:**
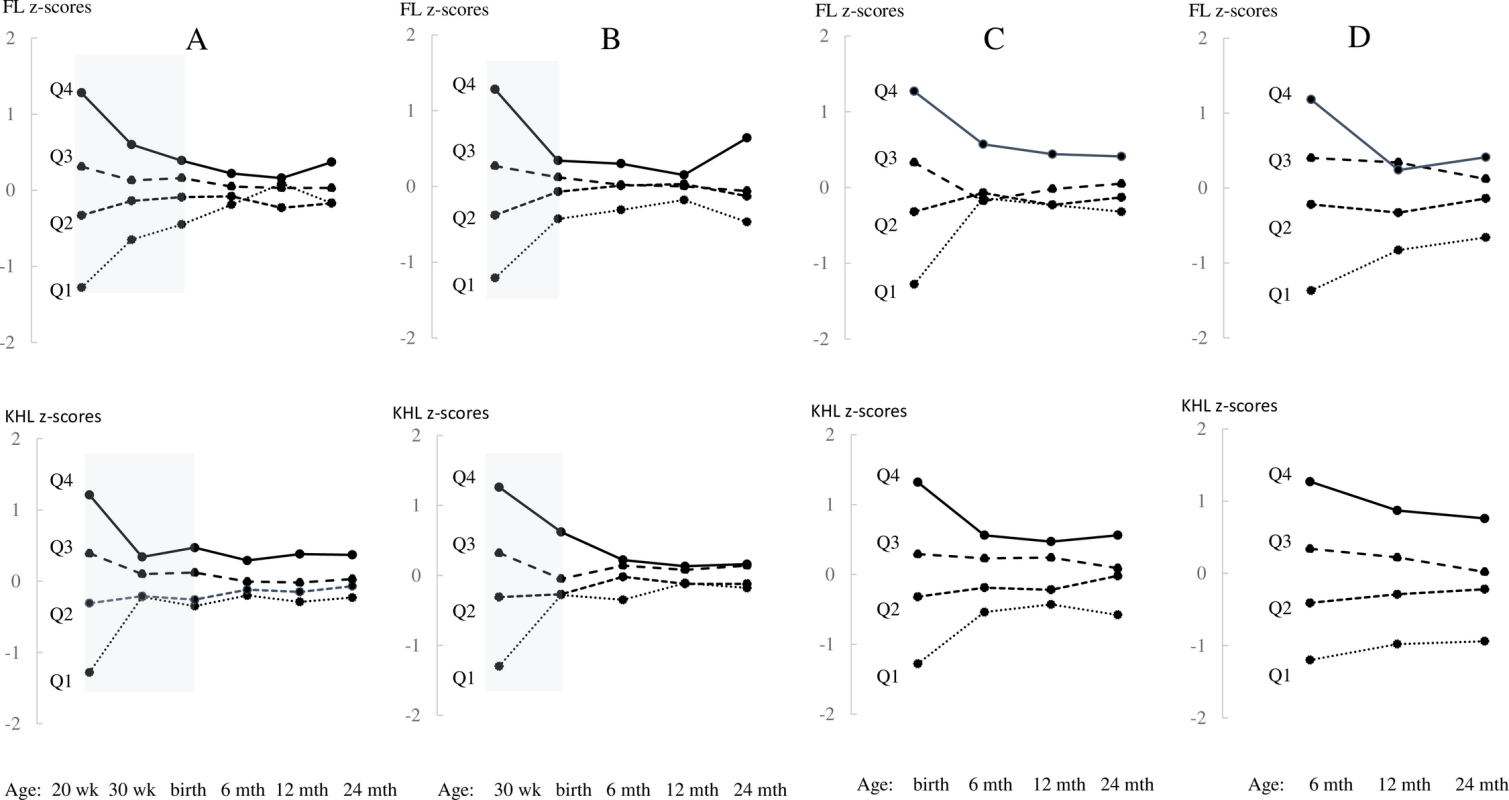
Offspring are classified according to quartiles of femur length (FL) and knee heel length (KHL) z-scores at 20 weeks gestation (A), at 30 weeks gestation (B), at birth (C) and at 6 months of age (D), and the resulting z-scores ranking is shown through 24 months of age. The antenatal period is marked in grey. Q1 = quartile 1 (solid line), Q2 = quartile 2, Q3 = quartile 3 and Q4 = quartile 4 (dashed lines).

When the offspring were classified according to quartiles of FL and KHL at 20 weeks gestation (A), at 30 weeks gestation (B), and at birth (C) the resulting ranking of FL and KHL quartiles converged and overlapped a lot (Fig 3). When classified according to quartiles of FL and KHL at 6 months (D) the resulting ranking remained more distinct through 24 months. At 24 months, 31% of the offspring had FL in the same quartile as at 20 weeks gestation, 26% had FL in the same quartile as at birth, and 36% had FL in the same quartile as at 6 months of age. At 24 months, 29% of the offspring had KHL in the same quartile as at 20 weeks gestation, 34% had KHL in the same quartile as at birth, and 45% had KHL in the quartile as at 6 months.

The foetal FL z-score at 20 weeks gestation did not predict the infant FL z-score at 12 months (β = 0.05 [-0.11, 0.21]) but did predict the FL z-score at 24 months of age (β = 0.18 [0.04, 0.32]) (Table 4). The foetal KHL z-score at 20 weeks gestation predicted the infant KHL z-score at 12 and 24 months (β = 0.19 [0.04, 0.34] and β = 0.17 [0.03, 0.31]). The neonatal FL, KHL, body length and body weight z-scores predicted the z-scores of the same traits at 12 and 24 months of age. The estimates were for FL: β = 0.23 [0.07, 0.39] and β = 0.33 [0.19, 0.47]; KHL: β = 0.30 [0.16, 0.44] and β = 0.32 [0.19, 0.45]; body length: β = 0.42 [0.29, 0.55] and β = 0.39 [0.27, 0.51]; and body weight: β = 0.31 [0.18, 0.44] and β = 0.29 [0.17, 0.41], respectively. Both maternal and paternal traits were associated with traits in infancy. The FL and KHL z-scores at 20 weeks gestation explained 4–5% of the variance in the ranking of the same trait at 24 months. The FL, KHL, body length and body weight z-scores at birth accounted for 13–26% of the total variance in the z-score of the same trait at 24 months. The parental KHL, body length and body weight accounted for 7–14% of the variance in the same traits at 24 months.

**Table 4:** Effect of offspring femur length and knee heel length z-scores in 20 weeks gestation and at birth, and parental same traits on subsequent offspring traits z-scores at 6 months, 12 months and 24 months of age.

	Overall	Gestation wk 30	At birth	At 6 months	At 12 months	At 24 months
Femur length (FL)	β (95% CI)	β (95% CI)	β (95% CI)	β (95% CI)	β (95% CI)	β (95% CI)
**FL gestation wk 20**		**0.46 (0.34, 0.58)**	**0.29 (0.17, 0.41)**	0.13 (-0.01, 0.27)	0.05 (-0.11, 0.21)	**0.18 (0.04, 0.32)**
**Maternal FL (SD)**	0.04 (-0.04, 0.12)					
**Paternal FL (SD)**	0.07 (-0.01, 0.15)					
**Neonatal FL at birth**				**0.27 (0.11, 0.43)**	**0.23 (0.07, 0.39)**	**0.33 (0.19, 0.47)**
**Maternal FL (SD)**	0.05 (-0.02, 0.17)					
**Paternal FL (SD)**	0.03 (-0.09, 0.15)					
**Knee heel length (KHL)**						
**KHL gestation wk 20**		**0.19 (0.08, 0.30**)	**0.28 (0.16, 0.40)**	**0.14 (0.00, 0.28)**	**0.19 (0.03, 0.35)**	**0.17 (0.03, 0.31)**
**Maternal KHL (SD)**	**0.15 (0.07, 0.23)**					
**Paternal KHL (SD)**	**0.17 (0.09, 0.25)**					
**Neonatal KHL at birth**				**0.33 (0.19, 0.47)**	**0.30 (0.16, 0.44)**	**0.32 (0.19, 0.45)**
**Maternal KHL (SD)**	**0.21 (0.11, 0.32)**					
**Paternal KHL (SD)**	0.07 (-0.07, 0.21)					
**Body length**						
**Neonatal birth length**				**0.39 (0.27, 0.51)**	**0.42 (0.29, 0.55)**	**0.39 (0.27, 0.51)**
**Maternal height (SD)**	0.11 (-0.01, 0.23)					
**Paternal height (SD)**	**0.21 (0.09, 0.33)**					
**Body weight**						
**Neonatal birth weight**				**0.22 (0.10, 0.34)**	**0.31 (0.18, 0.44)**	**0.29 (0.17, 0.41)**
**Maternal weight (SD)**	**0.18 (0.08, 0.28)**					
**Paternal weight(SD)**	0.06 (-0.05, 0.17)					

Values are β-estimates (95% confidence interval (CI)) per z-score change in offspring traits and per standard deviation (SD) change in parental same traits adjusted for maternal age, current smoking (no vs. yes), alcohol intake (no vs. yes), primiparous (yes vs. no), Caucasian ethnicity (no vs. yes), parental weight, and offspring sex using mixed models. Set p-value < 0.25 for entering variables and p-value > 0.15 for deleting variable.

## Discussion

Genetic factors determine most of the variance in height and bone dimensions in adulthood, and tracking is well documented during childhood and adulthood [2,11]. However, trait ranking in the first trimester only weakly predicted the ranking at birth [10,21]. We therefore examined whether intrauterine factors obscure genetic variance until 6 months postnatal, when the release of maternal constraints could restore the ranking achieved in early pregnancy. Specifically, we studied whether trait ranking in the second trimester predicts trait ranking in the first 1–2 years of life. Contrary to the hypothesis, the results did not show that ranking was established during gestation was lost and reinstated in the postnatal period. Tracking in skeletal size was robustly established 6–12 months postnatal. Trait ranking at 20 weeks gestation and at birth predicted trait ranking in infancy and explained up to 5% and 26%, respectively, of variance at 2 years. We confirmed the maternal influence on these traits, but there was no paternal influence on foetal dimensions during gestation. Dimensions of the same trait in both parents affected infant dimensions.

There have been few longitudinal studies relating antenatal growth to postnatal growth [7,22,23]. The work by Tanner suggests that maternal factors lead to a temporary reduction in foetal growth velocity in late pregnancy, and a tendency to revert to the original early foetal growth trajectory during the first 2 years of postnatal life in the context of sufficient nutrition and good health [12]. They further suggests that the increase in foetal body length decreases sharply after 30 weeks gestation, while the increase in foetal weight slows after 34 weeks gestation, constrained by placental nutrient flow and maternal pelvic size to enable successfully delivery [13]. In contrast, Harvey et al. reported that postnatal skeletal size at 4 years of age was more strongly associated with foetal dimensions and growth velocity in late pregnancy rather than in early pregnancy [22]. Our results tend to agree with this finding, as we did not observe any strong associations between foetal dimensions in early gestation and postnatal dimensions at 1 or 2 years of age. The mothers of infants lost to follow-up at 24 months of age were less often Caucasians in the current study. As the results differed little before and after adjustment for ethnicity included among covariates, this non-response is unlikely to have influenced the results.

Ounsted et al. suggested that maternal constraints slow foetal growth, even in normal pregnancies [24]. They concluded that the effects of maternal constraints are small and could be due to both genetic and environmental factors. Such effects were identified only in the lower extreme of maternal size, whereas in the upper extreme, the lack of constraints allowed other factors to determine more of the variance in foetal growth [24]. In contrast, Kuzawa et al. found no evidence supporting the hypothesis that maternal and paternal birth weight were stronger predictors of offspring birth weight when mothers were taller in a Philippines study population [25]. They therefore suggested that maternal effect on foetal growth are present across the whole range of maternal stature. Maternal constraint is not a well-defined term; there is paucity in the knowledge of the mechanisms underlying maternal constraints, and it is unknown whether such constraints operate across the whole range of foetal sizes, and the gestational periods [13] and have clinical implications.

Growth restriction can be identified early and late during gestation using ultrasound. Foetal FL in the first trimester, predicts antenatal and postnatal length, but the associations are stronger before birth than after birth [21]. Smaller foetal CRL in the first trimester led to compensatory accelerated postnatal growth, so there was no longer any association between foetal growth restriction and growth parameters at 24 months of age [21]. Foetal FL in the second and third trimester, is positively associated with body length/height in infancy and childhood [22,26,27]. Infants with smaller gains in length and weight in the third trimester have higher peak height and weight velocities after birth, but associations with catch-up growth in infancy are weak [27,28]. However, not all studies have reported that foetal growth restriction is followed by catch-up growth [29]. Interestingly, Harvey et al. reported that the relationship between growth parameters early in pregnancy are more strongly associated with bone parameters at 4 years, while growth parameters late in pregnancy are associated with bone traits more strongly at birth and less robustly at 4 years [30]. These previous results somewhat supported the hypothesis that maternal constraints can operate in late pregnancy, thus temporarily disturbing the genetically determined ranking of individuals with respect to size established in early pregnancy, and that this rank ordering will return in infancy. Taken together, the results indicate that the importance of antenatal and postnatal growth on health in childhood and adulthood is not clear.

Cooper et al. reported positive associations between birth size and bone mass and fracture risk in later life [8,31–33]. However, most of these associations were rather weak, and to the best of our knowledge, the variance in bone mass explained by birth size was not reported in these studies. Still, even small changes in trajectory of growth may change the ranking of the trait in adulthood [31]. In the current study, a small proportion (4–5%) of the individual ranking in FL and KHL at 2 years of age was explained by trait ranking at 20 weeks gestation, while ranking at birth explained 13–26% of the variance in trait ranking in infancy. While trait ranking during gestation and at birth correlated weakly or modestly with ranking in infancy, we found that strong correlations emerged for all traits after 6 months of age. This finding is in agreement with that reported by Wang et al., who reported that tracking in bone traits is established at 6 months postnatal, but not at birth [11]. They suggested that this could be partly the result of the release from maternal constraints, which permits expression of maternal, paternal and individual genetic factors during postnatal life [11], but we could not confirm this in the current study.

To the best of our knowledge, this is the first study of antenatal and postnatal growth of FL and KHL to include measurements of the same traits in both parents. Both maternal and paternal proportions are known to influence offspring proportions during postnatal growth [15]. This study confirms an effect of both parents on the same trait ranking in KHL, body length and body weight at 2 years, and this effect explained 7–14% of the total variance. Maternal, but not paternal, proportions predicted foetal proportions during gestation. Paternal data, which would only contribute to genetic influences on growth, may serve as a type of negative control. In a Norwegian study of more than 67,000 births, the correlation between maternal-offspring birth weight (r = 0.226) was stronger than the paternal-offspring birth weight (r = 0.126) [34]. Still, the causes for the stronger maternal than paternal contribution to birth outcomes are not well understood. Environmental, physiological, cultural, genetic or epigenetic factors can be difficult to separate, while false paternity are expected to play a small role [25]. This needs further research.

The strength of this study is the standardized research setting for obtaining measurements of both parents and offspring rather than using self-reported measurements of height and weight. However, there are some limitations to our study. The best way to assess age in the prenatal period, based on the first day of the last menstrual period, is prone to error because the interval from menstruation to fertilization varies from 8 to 20 days [12]. Although antenatal measurements were obtained by experienced ultrasonographers, and knemometer measurements are considered highly accurate, postnatal FL measurements are less accurate and influenced by the amount of subcutaneous adipose tissue at the bottom. Measurement errors may dilute true associations and lead to an underestimation of the effects, and the results must therefore be interpreted with caution. Another limitation is the lack of information on lifestyles, nutrition and food intake, which may influence postnatal growth and explain some of the variance in bone traits.

In conclusion, there was a small effect of foetal traits in the second and third trimesters and a larger effect of neonatal traits on traits at 2 years of age. These findings did not confirm our hypothesis that ranking is established early during antenatal growth, lost as a result of maternal constraints and reinstated during postnatal growth, thus ranking must be established later. Maternal, not paternal, influences were apparent during gestation, but both maternal and paternal parameters influenced the traits at 2 years of age as previously shown. Additional longitudinal studies of antenatal and postnatal growth are needed to obtain a better understanding of maternal constraints, the mechanism behind this phenomenon, and the long-term effects on offspring. Greater knowledge of the biological determinants of variation in foetal growth may be advantageous for decision-making regarding the need for intervention in clinical practice.

## Supporting information

S1 Dataset (SAS7BDAT)
